# Supplementary material for: Rollout of ShangRing circumcision with active surveillance for adverse events and monitoring for uptake in Kenya
Source: PLoS One. 2019 Sep 26;14(9):e0222942. doi: 10.1371/journal.pone.0222942 (PMC6762105; doi:10.1371/journal.pone.0222942)
Supplement: Attachment — Submitted filename: Response to Reviewers.docx [file pone.0222942.s002.docx]

**Pint-by-point Response to Reviewer Comments**

**Protocol title: Rollout of ShangRing circumcision with active surveillance for adverse events and monitoring for acceptability in Kenya**

| **No.** | **Comments** | **Response** |
| --- | --- | --- |
| **Journal Requirements** | | |
| 1 | When submitting your revision, please ensure that your manuscript meets PLOS ONE's style requirements, including those for file naming. | PLOS ONE style and file naming requirements addressed |
| 2 | Upon resubmission, please provide the following:   - The name of the colleague or the details of the professional service that edited your manuscript - A copy of your manuscript showing your changes by either highlighting them or using track changes (uploaded as a *supporting information* file) - A clean copy of the edited manuscript (uploaded as the new *manuscript* file) | We did not engage professional services to edit the manuscript. But we have acknowledged Renee Ridzon and Paul Musingila as professional colleagues who assisted with review and editing the manuscript.  Revised Manuscript with Track Changes is uploaded as a supporting information file.  Clean edited manuscript is uploaded as well.3 |
| 3 | In your Methods section and Ethics Statement, please specify whether data were obtained in a fully anonymized and de-identified manner, and whether any of the researchers/authors had access to identifying information. If any of the authors had access to identifying information (names, addresses, etc.), please explain whether you obtained participant consent, or whether the requirement for informed consent was waived.  Thank you for including your ethics statement: "This surveillance activity was reviewed by the Kenya ministry of Health VMMC program plus the Centers for Disease Control and Prevention (CDC), Center for Global Health (CGH) human research protection procedures and determined to be non-research (CDC CGH HSR Tracking # D-14-2015; 2016-173)."  a) Please amend your current ethics statement to confirm that your named institutional review board or ethics committee specifically approved this study.  Once you have amended this/these statement(s) in the Methods section of the manuscript, please add the same text to the “Ethics Statement” field of the submission form (via “Edit Submission”). | Data were obtained in routine clinical settings at public health facilities as part of the national VMMC program using a modified version of the national VMMC client form in which clients names and ShangRing-specific variables were recorded. We anonymized participants by using unique identifiers for analysis purposes. Authors did not have access to identifying information.  We obtained consent according to VMMC program standards, which require written informed consent before the circumcision regardless of the method. ShangRing-specific consent was obtained for each client circumcised through the device. Minors assented to the procedure but a parent or guardian granted consent for them.   a) We have amended the ethics statement in the manuscript body and added the same text to the ethics statement field of the submission form. |
| 4 | **Additional Editor Comments** | |
|  | 1) In response to reviewers, authors should state that conduct of the active surveillance was consistent with the WHO framework and similar, previous initiatives (e.g. Mavhu 2016; 2019). The working definition of "acceptability" is consistent with both WHO and similar, previous initiatives. Of note active surveillance is not a study per se. Other reviewers’ comments are however, relevant and need to be addressed. | Addressed in the manuscript |
|  | 2) Possible reasons for variability in acceptability (11-97%) need to be provided. In other instances, this was because providers were actively sabotaging a device as more VMMCs in a shorter time meant less remuneration. If possible reasons are unavailable, this should be stated as a limitation and future initiatives should explore these. If it was due to absence if systematic demand creation, a recommendation would be that device-specific demand creation should be intensified and/or target those sites/communities with low acceptance. | Addressed in the manuscript |
|  | 3) Also, future initiatives (including qualitative/mixed methods) should explore possible reasons for not taking up ShangRing - these will be critical in informing demand-creation for the device. | Addressed in the manuscript |
|  | 4) Active tracing procedures need to be described in detail (see previous device active surveillance papers) - How many call/text attempts and when? | Addressed in the manuscript |
|  | 5) Rest of comments are editorial - most have been raised by reviewers  .Authors vacillate between UK & US English (e.g. anesthesia/anaesthesia, program/programme, analysed) - should use one consistently  .References - 101, 104, 304, 333-334  .Line 71 - 12 years  .112-studies, (13)  .167 - of ShangRing?  .196 timely, (comma use)  .276 - is may?  .330 AES or AAES?  .347, 361 (10-12 years) | Addressed in the manuscript |
| **Response to comments by Reviewer No 1** | | |
| 5 | **Reviewer #1: Major comments:** This is an interesting paper on safety and acceptability of ShangRing in Kenya. It is clear that devices are an important part of VMMC scale up and efficiency. The paper would be of interest to your readers and is timely. The data could inform a critical component of HIV prevention programs.  However with the multiple purposes of this evaluation including assessment of ShangRing safety and acceptability, the authors fail to completely address that they could not demonstrably show acceptability. Safety was assessed and ShangRing demonstrated safe. An average acceptability of only 29% is poor – half of the 6 sites had acceptance rates of 25% or less. This result, in contrast to previous research, is diminished and ShangRing scale up repeatedly suggested, although premature based on these data. The abstract, discussion, and conclusions are, therefore, misleading. As acceptability was not demonstrated, the investment in training, devices, and alternatives to surgical MC for ShangRing roll out are not supported in this paper. Related, the results – only one sentence on acceptability (a major outcome) are not well balanced. It is explained in the methods and limitations that they did not collect information from clients on reasons not to want ShangRing as this was largely routine data collection; however, this major limitation should be better explained. Although they posit some potential explanations in a discussion paragraph, the lack of further exploration of this interesting result severely limits the utility of this research.  Attention and revision with an eye to the results on acceptability is needed to strengthen the paper. The authors should return to clinics, focal persons, or other key VMMC informants to illuminate these findings and give some further rational for this interesting result. This is the key piece of the paper that could inform scale in Kenya and the region. This would not take long and would help make the results more informative. | The conduct of the active surveillance was consistent with the WHO framework for evaluation of MC devices and similar previous initiatives (e.g. Mavhu 2016; 2019) now added as reference no 13.  Note that acceptability has been replaced with uptake in the title and body of the manuscript.  We observed wide variation in ShangRing **uptake** ranging from 11% to 97% across the six sites. Possible reasons include provider factors such as site level variation in demand creation practices despite existence of guidelines and actual difference client preference for the device across different communities. (E.g. fishermen from beach communities in Siaya have been more enthusiastic about ShangRing circumcision). The surveillance activity was conducted in the context of routine service delivery without recording each client’s reasons for choosing or not choosing ShangRing. We are unable to return to the clinic to investigate these findings therefore, we have stated this as a limitation and recommended that future initiatives explore reasons for such variations. Additionally we have recommended that device-specific demand creation be intensified and prioritized for communities where acceptance is low. |
| 6 | **Reviewer # 1: Minor comments:** Line 101: Fix references. This is distracting and poor editing: “other African counties, which demonstrated its safety, ease of use and good cosmetic outcomes [5, 6, 7, 8, 9 102 10, 11, 12], the Kenya national VMMC technical working group endorsed 103 its rollout under an active AE surveillance protocol in line with the WHO framework for clinical 104 evaluation of MC devices[Error! Bookmark not defined.].” Line 304/334 reference as well. | Thanks for catching these. Errors are corrected. |
|  | Line 114: Rephrase: It is not an “equivalent alternative.” This phrase seems misleading. | Line 114 rephrased to read …equally effective alternative… References edited accordingly. |
|  | Line 134: Please provide more details on the training ShangRing. How long was training? How was proficiency attained? How many procedures did the trainees watch or perform? Was AE identification, treatment, management, and documentation included? Please clarify. Evaluation sites were noted to be chosen for dense populations with persons living close to MC sites. However, you later note in lines 155+, that clients receiving ShangRing were transported from additional outreach intake points to the fixed sites. This seems to contradict your evaluation site criteria. | The training included a 2-day didactic session covering theory, AE management and documentation plus 1-day clinical observation of procedures performed by the trainers. Subsequently each trainee performed 8 placements and 8 removals to attain certification. The manuscript has been edited to reflect these details. |
|  | Line 161: This is the basis for the paper and needs more detail in this section. How was active surveillance conducted and when? Were phone calls the first line of active surveillance and then home visits? Or either? Or both? Were they triggered on Day 7 or Day 8? Please clarify the active tracing procedures. | According to the protocol, failure to return for device removal on day 7 or other scheduled appointments would trigger tracing action; starting with a phone call or short text message before close of business on the date of missed appointment. If contact is made, the client is re-scheduled for device removal within the shortest time possible at the facility or off-site at his home if necessary. If tracing by phone fails, physical tracing starts on day 9 and ceases on day 21 but client file remains open until the end of the surveillance activity. All clients who missed their removal appointments were successfully traced and removals completed at the designated static sites. |
|  | Line 190: Follow up rate outcome includes outcomes of active follow up efforts for clients who failed to return for follow up. Does this include only those with observed outcomes or also those who reported outcomes by phone? | Outcome of follow up efforts were documented as **reached** or **not reach** and the ultimate result would be returned for device removal or lost. All clients ultimately returned for device removal by day 11, hence no loss to follow up (shown in Fig 2). |
|  | 211: This is a large limitation to the usefulness of the study to inform scale up. “Reasons for choosing or declining ShangRing were not collected because this evaluation was implemented in routine service delivery settings.” | Yes, we acknowledge this limitation. The surveillance activity was conducted in the context of routine service delivery without recording each client’s reasons for choosing or not choosing ShangRing. We are unable to return to the clinic to investigate these findings therefore, we have stated this as a limitation and recommended that future initiatives explore reasons for such variations. Additionally we have recommended that device-specific demand creation be intensified and prioritized for communities where acceptance is low. |
|  | Table 2: Why do you think that Mbita District Hospital had the majority of the missing ShangRIng sizes? | Mbita may have been more stringent in ensuring that the device size used perfectly matched the penile size measurement to the last millimetre. Less stringent sites may possibly have more flexible with device selection, possible replacing missing sizes with the next 1mm smaller or larger size. |
|  | 238: The fact that acceptability of ShangRing varied widely is suggestive of highly varied recruitment or demand creation procedures. How were demand creation officers trained? How did that training vary across sites? Were the mobilizers paid to recruit? Was that the same across sites? |  |
|  | 259: No need to repeat outcome measures of timing. | Addressed. |
|  | Line 267 spelling error, “avialble” is an example of the need for grammar, spacing, and spelling review throughout. There are many examples of sloppy copy editing. Another: Line 276, “and age is may have been obscured due to the small number of clients in this age bracket.” Line 330 “experienced during this AES.” Even ShangRing is not consistently spelled throughout (check your tables and figures). Many more missing punctuation and errors to be addressed that show lack of close editing review.  Results lines 300+. This lower acceptability seems like the major result. This paragraph could be strengthened. What different in lower and higher acceptance sites? Just urban/rural? Was it only lack of ShangRing availability at the sites? If the transportation was a factor, this also shows that the sites were poorly selected as the criteria for inclusion suggested that close proximity to sites was a reason for site selection. Please explain. | Demand creation officers for all the 6 activity sites attended the same training. Mobilizers were employees paid a fixed monthly salary. This activity was implemented in routine program implementation context rather than a study. Consequently, the observed variations could be a reflection of site-level deviations from prescribed demand creation practices in the program.  Spelling errors are noted and corrected.  ShangRing being a newly introduced device that was presented as an equally effective alternative to the existing standard (conventional surgery), an initial low acceptability is not entirely surprising. Uptake is likely to change (increase or even decrease) as more people get to know it better. We recommend that device-specific demand creation be intensified in sites or communities with low acceptance/uptake. Other important findings include;   1. Re-affirmation of device safety based on a larger number of procedures 2. Absence of new AEs (all observed AEs had been experienced and described in earlier studies) 3. Minimal operational bottleneck (lack of some device sizes at the time of procedure was the main one) 4. Low rates of clinical ineligibility for the procedure-consistent with earlier studies |
| 7 | **Response to comments by Reviewer No 2** | |
|  | Reviewer #2: Thank you to the authors for the manuscript that focuses on the active surveillance of the Shang Ring device in Kenya across 6 sites in 5 counties. While I consider the manuscript to be valuable it does require some revision and points of clarity.  Abstract 1. Conclusion in the abstract reads as a direct copy of the main manuscript conclusion. | Abstract 1. Conclusion in the abstract conveys the same message as in the main manuscript conclusion but has been edited and now reads differently. |
|  | Background 1. Where it is written medical circumcision recommend amending to read medical male circumcision (MMC) to be specific 2. There are sentences that require references and reference correction: end of sentence line 88, end of sentence line 92, end of sentence line 94, end of sentence line 97, reference 9 in line 102, reference error in line 104. 3. The authors mention that the goal of the surveillance activity was to “assess the feasibility of Shang Ring…” however, as per the WHO guidelines on evaluating medical male circumcision devices, feasibility is usually assessed during the pilot stage. Therefore, was this a true feasibility assessment? Furthermore, it is not clear what the operational challenges or opportunities were within the broader scope of the manuscript. 4. Were the outlined objectives representative of the overall active surveillance or the manuscript? Recommend revising these objectives to outline the aims of the manuscript under background and the active surveillance objectives can be included under the methods section. However, as it stands the presented objectives do not speak to the results of the manuscript. An example: objective (2) “detect new or rare AEs…” nothing new or rare was reported. None of the objectives clearly talk to operational challenges or operational bottlenecks. Based on the discussion, one can make the assumption that objective 4 makes reference to operational challenges however the link is not clear. | **Background**  1. medical circumcision (MC) replaced with medical male circumcision (MMC)  2. Additional references included and corrections made  3. We postulated that every phase of device evaluation has a feasibility assessment component; each of which contributes to the body of evidence for final decision making on large-scale roll out of the device. The initial pilot demonstrated the feasibility of ShangRing circumcision roll out and absence of serious operational bottlenecks based on a small number of procedures.  4. The active AE surveillance presented an opportunity to further evaluate safety, feasibility of roll out, uptake and detect any rare adverse events based on a larger sample size. With the larger sample size, we had higher likelihood of detecting rare AE that may have been missed in the pilot phase (due to small sample size). Results 1: ShangRing circumcision remained safe based on larger sample size (Moderate/ severe AE rate remained low) Result 2: Out of the larger sample of over 1000 procedures we did not come across any adverse event that had previously not been described Result 3: We identified an operational challenge associated with the need to stock large numbers of different device sizes (some sites missed the required device size at the time of the procedure) Result 4: Wide variation in uptake probably influenced in part by differences site level demand creation practices and possible need to transfer ShangRing clients from outreach to static sites for facilities. |
|  | **Methods** 1. Were the health facilities or counties supported by PEPFAR. Consider re-wording for clarity. 2. Selection criteria for sites: the authors list 3 however it reads as 4. Population density and travel time should be two separate criteria. 3. What was considered to be a ‘short’ travel time between site and residence? 4. How did the authors define a ‘competent’ healthcare worker in conventional surgical MMC? 5. The target was 167 per site, however some were under and others over? Was the target enrolment controlled across sites? Did all sites start initiation of the Shang Ring at the same time? 6. Under training, the authors touch on sensitization of non-participating health care workers and the general public how was this achieved, through workshops, information sessions, pamphlets… 7. The study only enrolled HIV uninfected clients, does this mean that clients were tested at the facility prior to being offered the Shang Ring or was HIV status considered under screening? 8. Line 146 to 149 is unclear, recommend rephrasing to: Clients who chose ShangRing but were found clinically ineligible for the device (due to conditions like adhesions and thick or short foreskins) while eligible for surgery were circumcised through conventional surgery according to the Kenya clinical manual for male circumcision under local anaesthesia (13). 9. Under procedure it mentions that clients consent to “active follow-up” however the study does not continue to follow clients after device removal. This needs to be clarified. 10. Provide additional information to explain why some clients had to be transported to a service delivery point to receive the Shang Ring. Was this the same at those sites for clients opting for conventional MMC? 11. How was wound healing determined? 12. There is no description of how the data was collected and captured. Were these clinic files that were reviewed daily, logs that were assessed retrospectively and where was the information captured and stored?  Outcome measures 13.1. Proportion of AEs was calculated by adding the severe and moderate not severe or moderate 13.2. Under outcomes: safety is reported by the AE rate, acceptability is reported by device uptake. However, it is not clear how clinical eligibility, effectiveness, follow-up rate, device placement duration are related to operational challenges. 13.3. Does time until return for removal fall under follow up rate? This is confusing considering that the study has no formal follow-ups  14. Data analysis section should be a separate paragraph 15. Provider experience as a measurement only features under analysis description and does not appear in the results, discussion or background. Not clear how it is relevant. | **Methods**   1. PEPFAR supports county specific implementing partners who in turn support Health facilities in the target counties (support covers both county and facilities). 2. Population density and travel time to health facilities are interlinked and were applied broadly to ensure reasonable level of access to ShangRing for the target population. Segregating them as this point may not change the results of the surveillance activity. 3. Short travel time is an indicative measure of the ease of accessing service delivery; cut off point is 5km; 4. Healthcare workers competent in conventional surgical MMC are those trained and certified according the Kenya national guidelines for VMMC. 5. Sites did not all start at the same and accrual rates varied across them but there was provision for pooled analysis of data. Assignment of about 167 procedures per site was for planning. 6. Clinical personnel in non-participating health facilities in the catchment areas of the implementing sites were oriented on ShangRing and AE management. The goal was to ensure appropriate management and collection of accurate information on ShangRing clients present to their site. Guidelines on assessment, referral and management of ShangRing clients was provided at facility sensitization meetings lasting half a day. 7. Only HIV negative males aged 13yrs and above. Testing for HIV was done at the site. This is in line with WHO framework for clinical evaluation of MC devices. ShangRing for HIV+ve clients falls under the scope of bridging studies. 8. Thanks for catching this. Line 146-149 edited as suggested. 9. Only one follow up was scheduled for device removal on day 7 in line with overall VMMC program follow up guidelines. Active follow up was limited to tracing clients who missed their appointment for device removal on day 7 or any other scheduled contact. This surveillance was conducted in the context of routine program implementation. Prolonged and meticulous follow up until complete wound healing would be a duplication earlier pilot studies. 10. For some AAES sites, in addition to providing surgical VMMC and ShangRing at the fixed site, providers conducted outreach VMMC services as part of their ongoing service delivery obligation under PEPFAR funding. Consequently the site would provide surgical MMC at fixed site and at outreach points. But ShangRing circumcision was only available at the fixed site. Therefore, any ShangRing clients identified at the outreach points were transported to the fixed site. This did not happen across all sites may partially explain the observed variation in uptake across sites. 11. This AAES activity did not include assessment of wound healing but provided a description of completely as part of client education. Healed wound is dry with apposed edges and no gaps or scabs. 12. Data were obtained in routine clinical settings at public health facilities as part of the national VMMC program using a modified version of the national VMMC client form in which clients names and ShangRing-specific variables were recorded. We anonymized participants by using unique identifiers for analysis purposes. All other aspects of data handling conformed to national VMMC program guidelines. 13. (13.1)We counted the number of unique individuals who had moderate or severe AES as numerator and total number of clients who underwent ShangRing procedure as the denominator. 14. (13.1) clinical eligibility – would pose a challenge for device roll out if too many clients who are HIV-ve, are 13 or more and chose ShangRing were found to be clinically ineligible for ShangRring   Effectiveness- the foreskin was successfully excised in virtually all ShangRing procedures. Frequent failures of ShangRing procedures would pose a serious operational challenge because surgical completion with suturing would be required in all cases of failed device circumcision. Device placement duration would pose operational challenges and undermine feasibility if it is too long. Time saving is a ke selling point for ShangRing.   15. (13.3) Follow up rate is the proportion of clients turning up for their appointments. It is measured by time pint since the procedure. 100% day 7 follow up success for ShangRing would be ideal. But this was not the case. 100% was attained at day 11. 16. (14) Data analysis is moved to a separate paragraph   Provider experience as a measurement and analysis was not deemed necessary for addressing the objectives of this AAES |
|  | **Results** 1. The study dates in the abstract are different from the dates in the results 2. In line 211-212 authors provide justification for why acceptability data is not collected, this is misplaced and should be moved from results to methods. 3. Figure 1: 11 clinically ineligible with not description in figure for why however 17 circumcised through conventional surgery with a description for why. Be consistent with how you represent your information. 4. What happened to the other 6 who were ineligible for Shang Ring and conventional surgical circumcision, were they referred for care? 5. Move sentence “Ten out of the 11 clients who were…” (line 222-223) before sentence starting with “Seventeen (1.6%)…” (line 220) 6. Be consistent with decimals when reporting percentages 1.6% vs 97% 7. It is not clear what the authors mean by “There were no previously undescribed ShangRing related AEs” (line 250-251) 8. What is the significance on reporting on duration of device placement? Device placement is not included in the discussion section. Furthermore, there is no data provided on duration of device removal. 9. Check Table of ShangRing device sizes: in table, A4 is 36 mm while in text A4 is 40mm. 10. Figure 3: The distribution of device sizes by age using the scatter plot is good. As the authors describe the plot is biphasic (13-20 years) and 20+ years regarding distribution of need for different device sizes. Device size variability is required in the younger age categories. | **Results**   1. The study dates in the abstract and in the results section have ben aligned (2106-2018). 2. Justification for why acceptability data is not collected has been moved from results to methods. 3. Reasons for clinical ineligibility were multiple and would not fit within the text box. The 17 circumcised through conventional surgery had a single common reason included. 4. 6 who were ineligible for Shang Ring and conventional surgical circumcision, were they referred for management of the underlying clinical conditions. 5. Suggested edit done. 6. Decimal place issues addressed 7. All observed AEs in AAES had previously been experienced and characterized in the early phases of ShangRing introduction in Kenya. This was a stated objective of the activity. 8. Duration of device placement the most important determinant of time saving associated with ShangRring Circumcision. 9. Aligned device size A (40mm) in Table of ShangRing device sizes and in text. 10. This is acknowledged. |
|  | **Discussion** 1. Consider re-working the sections of the discussion to follow the order in which the results are reported. Current order of results: Eligibility, Acceptability, AE, Placement duration, follow-up rate, size distribution. The discussion sections are: Eligibility, AE, Acceptability, Size. 2. The authors spend a considerable length of the discussion justifying the reasons for not collecting additional information on acceptability for this reason there are certain sections of the discussion that read like they should be under the ‘Limitations’ heading. A recommendation would be to change the wording from acceptability and instead report on uptake of the ShangRing device with a future recommendation for more detailed acceptability studies to be done. 3. The discussion section focuses too much on the 17 cases that did not have the appropriate device sizes available. However, there is no description in the results of what these sizes were or the age categories of the clients. The manner in which the device size is represented in the discussion section renders the scatter plot irrelevant. Recommend finding a way to report on the sizes that were relevant to the active surveillance. 4. The discussion section would benefit from additional literature to substantiate some of the points being made by the authors. | **Discussion edited to address review comments.** |
|  | Limitations 1. The description of no additional visits should also be reflected in the | Limitations  Included in methodology and listed as a limitation for detection of let onset AEs. |
|  | Conclusion 1. Remove references from conclusion 2. The WHO amendment to the prequalification is mentioned in the Limitations and Conclusions section however it is not in the Discussion section. Its link to the overall paper is not clear. Acknowledgement 1. Acknowledge the clients who participated in the active surveillance | **Conclusions edited to address review comments** |
|  | 1. Acknowledge the clients who participated in the active surveillance | Edited to include acknowledgement of participants |
