# Supplementary material for: Rollout of ShangRing circumcision with active surveillance for adverse events and monitoring for uptake in Kenya
Source: PLoS One. 2019 Sep 26;14(9):e0222942. doi: 10.1371/journal.pone.0222942 (PMC6762105; doi:10.1371/journal.pone.0222942)
Supplement: Attachment — Submitted filename: Response to Reviewers.docx [file pone.0222942.s003.docx]

**Pint-by-point Response to Reviewer Comments**

**Protocol title: Rollout of ShangRing circumcision with active surveillance for adverse events and monitoring for uptake in Kenya. PONE-D-19-15563R1**

| **No.** | **Comments** | **Response** |
| --- | --- | --- |
| **Additional Editor Comments** | | |
| 1 | Line 139- Subsequently, | Edited as suggested |
| 2 | 182 - ...who chose... | Edited as suggested |
| 3 | 260 - ...third AE, also moderate, was... | Edited as suggested |
| 4 | 270 Removals were... beyond). repeats lines 206-209 - could be paraphrased or left out | Paraphrased |
| 5 | 326 ...locations,...?? | Edited as suggested |
| 6 | 366 ...uptake of ShangRing... | Edited as suggested |
| 7 | 382 ....realization of public... | Edited as suggested |
| 8 | 384 ...adolescents 10-12 years... | Edited as suggested |
